# A Rare Case of Streptococcus cristatus Spondylodiscitis Identified by Bacterial 16S rRNA Polymerase Chain Reaction Sequencing: A Case Report and a Review of the Literature

**DOI:** 10.7759/cureus.59127

**Published:** 2024-04-27

**Authors:** Damien Dillie, Laurence Bamps, Maria Angeles Argudín, Hector Rodriguez-Villalobos, Thomas Kirchgesner, Edward Fomekong, Jean Cyr Yombi

**Affiliations:** 1 Internal Medicine and Infectious Diseases, Cliniques Universitaires Saint Luc, Brussels, BEL; 2 Microbiology, Cliniques Universitaires Saint Luc, Brussels, BEL; 3 Medical Imaging, Cliniques Universitaires Saint Luc, Brussels, BEL; 4 Neurosurgery, Cliniques Universitaires Saint Luc, Brussels, BEL

**Keywords:** molecular techniques, osteoarticular infections, pcr 16s rrna, streptococcus cristatus, spondylodiscitis

## Abstract

We report here a rare case of spondylodiscitis due to* Streptococcus cristatus *in a healthy 66-year-old male. Due to an abscess causing neurological deficit, which required immediate surgical intervention, a PCR targeting 16S rRNA was performed on the surgical samples as all blood and tissue cultures remained negative. This molecular assay allowed for the identification of this rare *Streptococcus*, a member of the mitis group and commensal of the oral cavity, whose pathogenicity remains uncertain although it has been seldom reported in cases of human infections, mostly bacteremia and endocarditis. Notably, our case is distinguished by the absence of comorbidities, although the patient’s history was compatible with a dental portal of entry. This case illustrates once more that 16S rRNA PCR can be of great help for documenting the causative pathogen in osteoarticular infections when cultures remain inconclusive. We reviewed in this article the data regarding osteoarticular infections due to *S. cristatus* and discussed the role of molecular technique in the diagnosis of spondylodiscitis.

## Introduction

Spondylodiscitis or vertebral osteomyelitis is an infection of both the intervertebral disc and the adjacent vertebrae that can occur from either hematogenous spread or direct inoculation by spinal surgery or spread from nearby soft tissue infection [1]. The most common cause is by hematogenous spread of microorganisms following a primary or secondary bacteremia, whether it is transient (e.g., after dental procedures) and physiological (e.g., after tooth brushing) or sustained in the case of an infection (including endocarditis). Known predisposing factors are sex (men), age, diabetes, immunosuppression, intravenous drug use, a history of recent injury or trauma, or invasive procedure [1]. 

*Streptococcus cristatus* is a gram-positive, catalase-negative cocci member of the* Streptococcus mitis* group and commensal of the oral cavity. It has been reported to cause bacteremia and endocarditis [2-10]. However, its pathogenicity in osteoarticular infections is less well understood and, to our knowledge, only two cases have been described: one case of septic arthritis of the wrist in a neonate and one case of vertebral osteomyelitis in an adult patient [9,10]. We report here the second case of spondylodiscitis due to *S. cristatus*.

Managing these complicated infections requires identifying the causative microorganism to determine the appropriate antibiotic therapy. False negative cultures can occur due to the presence of bacteria that grow slowly or are challenging to cultivate, but mostly because of antibiotic administration at the time of sampling. Molecular analysis has emerged as an additional diagnostic tool that is particularly useful when traditional cultures remain negative.

## Case presentation

A 66-year-old male with no prior medical history was admitted to the emergency department due to severe axial low back pain worsened with movement.

Twelve days earlier, the patient experienced chills but did not measure his temperature. The next day, he woke up with intense pain in the lower back. He did not notice either sensory impairment or saddle anesthesia but had remained bedridden due to severe pain for the past 10 days. On the day before his admission, he also reported a single episode of urinary incontinence. He denied any weight loss or other systemic symptoms. He reported no history of recent trauma, but he was involved in a road accident two years prior, resulting in significant back pain that had resolved since then. He had returned from Brazil two months and a half prior, where he had spent four weeks in the Goiás region. He reported no contact with animals. Upon his return, he went to his dentist and underwent a teeth scaling two months before his presentation.

On admission, vital signs were as follows: a blood pressure of 150/75 mmHg, a temperature of 36.1°C, heart rate of 79/minute, and oxygen saturation of 98% on room air. On clinical examination, a painful vertebral percussion at the L3-L4-L5 level was noted, along with an isolated motor deficit of the right psoas muscle (3/5 strength on the Medical Research Council (MRC) scale) with normal sensation [11].

Blood investigations revealed an elevated C-reactive protein (CRP) at 63 mg/L (normal range: <5 mg/L) with neutrophilic hyperleukocytosis (8170/µL, normal range: 1600-7000/µL), thrombocytosis (494 000/µL, normal range: 150 000-400 000/µL), and elevated creatine phosphokinase (240 U/L, normal range: 20-200 U/L). Urine screening was negative. Three sets of aerobic and anaerobic blood cultures were obtained.

**Figure 1:**
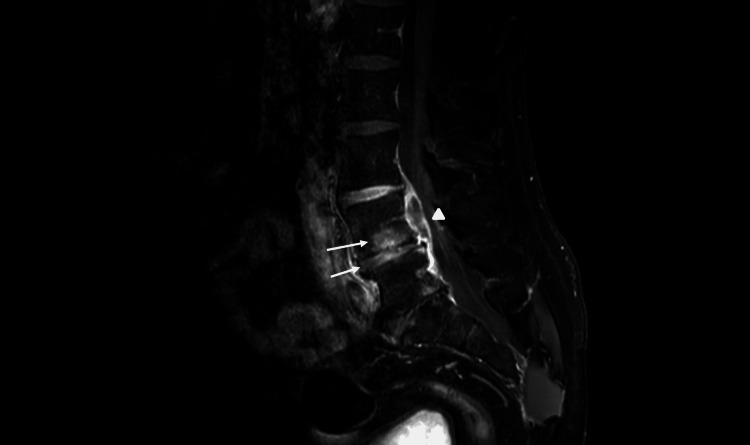
Sagittal fat-suppressed T1-weighted MRI after gadolinium injection Abnormal enhancement of the L4 vertebral body and the L4-L5 disc (arrows) with an anterior epidural abscess measuring 3x1x1.8 cm (arrowhead) MRI, magnetic resonance imaging

**Figure 2:**
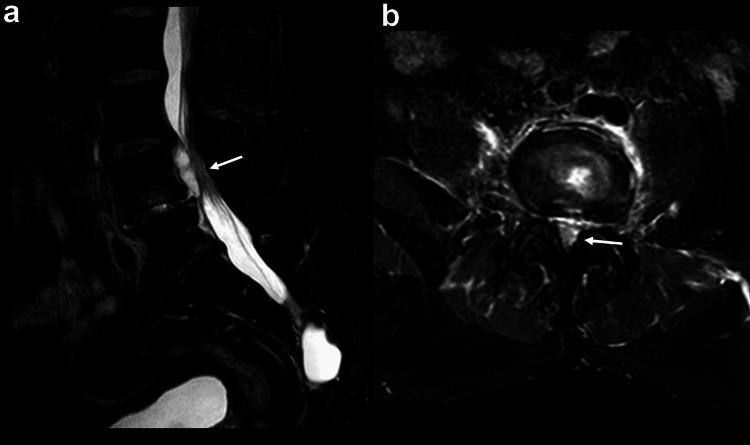
Sagittal (a) and axial (b) fat-suppressed T2-weighted MRI Severe canal stenosis at the level of L4 due to the collection, with an anteroposterior diameter of the dural sac measured at 4 mm and loss of the visibility of the rootlets and cerebrospinal fluid. MRI, magnetic resonance imaging

Given the high clinical suspicion of spondylodiscitis, magnetic resonance imaging (MRI) of the lumbar spine was performed and revealed signs of L4-L5 spondylodiscitis associated with a stenosing epidural abscess measuring 3x1x1.8 cm and expanding into the spinal canal at L4 level (Figures 1, 2).

The patient was hospitalized in our internal medicine ward for further investigations without empirical antibiotic therapy. Due to the presence of an abscess, intractable pain, and acute-onset neurological deficit, the patient underwent the minimally invasive neurosurgical procedure with L4-L5 disc biopsy and drainage of the epidural abscess. Samples were sent for bacterial, fungal, and acid-fast bacteria cultures and for histopathological examination.

Unfortunately, Gram staining was negative and no microorganisms were detected either in the cultures of the surgical samples or in the blood. Pathology on the disc samples revealed an acute inflammatory synovium, consistent with the diagnosis of spondylodiscitis. A transthoracic cardiac echography did not show any signs of endocarditis. Serology tests for* Brucella* and *Coxiella burnetti* were negative as well as the Interferon-gamma release assay for *Mycobacterium tuberculosis*.

Therefore, an amplicon-based 16S subunit rRNA) sequencing was performed on one of the surgical samples. Prior to DNA extraction, the biopsy sample underwent complete tissue lysis by incubation in 180 μL ATL buffer (QIAGEN) and 20 μL proteinase K (QIAGEN) at 65°C for 30 min with shaking at 900 rpm followed by an incubation at 95°C for 10 minutes. DNA was extracted using the ELITe InGenius® SP 1000 kit on the ELITe InGenius® instrument (ELITechGroup, Puteaux, France). The 16s rRNA sequencing has been performed in two PCRs by combining the 27F-YM+3 combination with the universal reverse primers 1522R and 1492R [12]. Amplicons were sequenced using the platform 3500xL DX Genetic Analyzer (Applied Biosystems, Carlsbad, CA, USA) with the BigDye 3.1 Terminator kit (Applied Biosystems). Sequence analysis was performed with the software Geneious Prime 2023. The consensus sequence was submitted to the EZBioCloud and Basic Local Alignment Search Tool (BLAST) databases for species identification. The sequence obtained (Accession No. PP659729) showed a high concordance with the *S. cristatus* reference strain ATCC 51100 at both EZBioCloud (99.71%; Accession No. GL732519) and BLAST (99.50%; Accession No. NR_042771.1) databases.

Immediately after the surgery, the patient was started empirically on high-dose IV ceftriaxone (2 g bid) according to our institutional practices for non-prosthetic acute osteoarticular infections until the causal pathogen was identified. After 13 days of IV therapy, an oral switch to levofloxacin (500 mg bid) was made upon discharge for a total of six weeks. This antibiotic was chosen for its good bioavailability, excellent tissue penetration, and the usual susceptibility of *Streptococci* to it (in the absence of a susceptibility test available).

He was followed up at the outpatient clinic at two and six weeks and reported an overall improvement in his symptoms; CRP was virtually normalized (6.9 mg/L, normal range: <5 mg/L).

## Discussion

Spondylodiscitis remains a challenging diagnosis. Symptoms can be nonspecific, and fever is poorly sensitive (present in only 52-68% of cases) [1,13]. Focal back pain is the predominant physical examination finding, observed in approximately 86% of patients, and the lumbar area is most commonly affected (58% of the cases) in bacterial spondylodiscitis [13]. Studies report an incidence of neurological compression in 33-59% of patients [1]. The presence of neurological symptoms or signs should alert the clinician to consider the possibility of a mass effect caused by an abscess in the epidural space. The reported prevalence of abscess formation varies but is documented in the literature as ranging from 35% to 74% [1].

Imaging is of great assistance in diagnosing spondylodiscitis, as well as in evaluating its complications. MRI provides greater sensitivity than CT scans, thereby allowing an earlier detection of lesions [1,14].

While hematogenous dissemination is the primary source of infection, blood cultures can yield negative results. It is crucial to bear in mind that it is frequently transient bacteremia, rather than overt bacteremia, that plays a role in infecting the vertebrae. Therefore, the reported prevalence of positive blood cultures ranges from 55% to 75% [1]. While not sufficient for confirming the diagnosis, routine blood culture testing is recommended as it can provide valuable guidance for antibiotic therapy, thereby avoiding the need for an invasive diagnostic procedure. When blood cultures remain negative or a polymicrobial infection is suspected, a biopsy (first percutaneous, and if still inconclusive on two occasions, surgical) is required, with CT-guided biopsies yielding positive results in 50% to 79% [1,14]. It is important to note that culture from biopsies conducted prior to the antibiotic treatment identifies a causative organism in approximately 75%-80% of cases, whereas biopsies conducted after the initiation of antibiotics are less sensitive (identification in around 50%) [15]. This emphasizes the importance of withholding antibiotic treatment until a causative microorganism is identified if the patient's condition allows it.

*Streptococci* are recognized as the third most frequent cause of spondylodiscitis (range 5% to 20%), after *Staphylococcus aureus* (accounting for half of the non-tuberculous cases, range 20% to 84%) and gram-negative bacteria (range 7%-33%) [16]. A Spanish prospective study demonstrated that spondylodiscitis caused by oral *streptococci *(formerly grouped within the *viridans *group) had a subacute presentation, leading to later diagnoses [17]. They were also more frequently associated with endocarditis [17].

The pathogen identified in this case, *S. cristatus*, is an oral *Streptococcus *member of the *S. mitis* group. It was first characterized in 1991 as gram-positive cocci, catalase-negative, in a 1 μm chain [18]. In 2014, a study based on the clustering patterns resulting from phylogenetic analysis of the complete genome of several species of the mitis group concluded that *S. cristatus *was closely related to *Streptococcus oligofermentans* [19]. Phylogenetic investigations also revealed a close relationship between *S. oligofermentans* and *S. sinensis *[20]. As a result, a novel phylogenetic clade, the "*sinensis group*", has been suggested to encompass *S. sinensis, S. oligofermentans,* and *S. cristatus* [20].

Similarly to other members of this group, *S. cristatus* is mainly found on mucosal surfaces of the oral cavity. Yet, there is limited knowledge regarding its pathogenicity. Since phenotypic identification of *viridans streptococci* to species level is based on colony morphology, growth characteristics, and biochemical reactions, infection with this species is probably underreported in the case of polymicrobial infections.

We found to date 13 other clinical cases of infections caused by this microorganism in the literature (Table 1). Among these cases, seven involved infective endocarditis [2-7]. However, in some of these cases, the co-existence of an additional microorganism raises uncertainty about the actual pathogenicity of *S. cristatus* [2]. The other reports describe cases of bacteremia, one case of peripheral septic arthritis in a neonate, one case of endophthalmitis after glaucoma surgery, and one case of vertebral osteomyelitis [2,8-10]. Most patients had comorbidities and/or poor dental hygiene, characteristics our patient did not present. Nonetheless, he had undergone a dental procedure, which could have caused the bacteremia. The hypothesis of a dormant infection following the history of road accidents seems unlikely given the delay of over two years, but any vertebral injury could lead to an increase in local vascularity and facilitate the hematogenous spread to vertebral bodies [1].

**Table 1:** Cases of Streptococcus cristatus infections IE, infective endocarditis; HD, high dose

Article	Patient profile	Source of infection and identification	Diagnosis	Coinfectants	Complications	Treatment
Matthys, 2006 [2]	37-year-old immunocompetent male	Blood cultures	Bacteremia and IE	Streptococcus mitis	Severe aortic insufficiency (grades 3-4) requiring prosthetic valve	Ampicillin 2 g 6x/day and gentamycin 1.5 mg/kg 3x/day and valve replacement
Matthys, 2006 [2]	52-years-old epileptic male	Resected aortic valve, culture	IE	Coagulase-negative* Staphylococcus*	Severe aortic and mitral insufficiency, mitral perforation, and left-sided heart failure	Failed: ceftriaxone + metronidazole; succeeded: ampicillin 2g 6x/day + gentamycin 60 mg 3x/day, then piperacillin-tazobactam 4g 4x/day + gentamycin, then ampicillin HD then oxacillin 2g 6x/day + vancomycin
Matthys, 2006 [2]	3-year-old female with a history of mental retardation and epilepsy	Blood cultures	Transient bacteremia	-	-	Amoxicillin-clavulanic acid
Isaksson, 2015 [3]	2 patients, no information	Blood cultures	Bacteremia and IE	-	No information	No information
Bele, 2020 [4]	57-year-old male with neurofibromatosis type 1 and mitral valve prolapse	Blood cultures	Bacteremia and IE	-	ANCA-PR3 positive immunocomplex glomerulonephritis	Penicillin. Mitral valve repair with resection
Gupta, 2020 [9]	15-day-old immunocompetent male	Synovial fluid of the wrist, culture	Septic arthritis of the wrist	-	-	Vancomycin for 4 weeks. Aspiration followed by arthrotomy
Lieberman, 2021 [5]	14-year-old female with 22q11 deletion syndrome and Tetralogy of Fallot	PCR 16s on pulmonary valve	Bacteremia and IE	-	Pulmonary valve replacement	Cefepime + vancomycin + gentamicin replaced after with ceftriaxone + daptomycin
Choe, 2021 [21]	59-year-old woman with history of glaucoma	Vitreous fluid, culture	Postoperative endophthalmitis	-	Left-sided bleb-related endophthalmitis and permanent left-sided vision loss	Topicals tobramycin, cefazolin, and moxifloxacin; systemic: cefuroxime 1.5 g/day and tobramycin 160 mg/day
Vecilla, 2022 [6]	72-year-old male with a history of bilateral pulmonary thromboembolism and biological aortic valve replacement 20 years earlier due to severe aortic insufficiency	Blood cultures + PCR 16s on the resected valve	Bacteremia and IE	-	Splenic infarction severe aortic insufficiency requiring prosthetic valve replacement	Piperacil­lin-tazobactam then ceftriaxone for 6 weeks + valve replacement
Vecilla, 2023 [7]	76-year-old male with a history of aortic valve replacement and partial hepatectomy due to liver cirrhosis with hepatocarcinoma	Blood cultures	Bacteremia and probable endocarditis	-	Small infarcts in the lower pole of the spleen and left kidney were found	Vancomycin (1 g/12h) and cefepime (2 g/24 h) replaced by Ceftriaxone
Guzman, 2023 [8]	59-year-old male with a history of end-stage cryptogenic cirrhosis and ascites	Blood cultures	Bacteriema	-	-	8 days of ceftriaxone, transitioning to cefpodoxime for 6 days after discharge
Tran, 2023 [10]	65-year-old male with a history of myasthenia gravis, hypertension, chronic kidney disease, and diabetes	Spinal biopsy, PCR 16s	Vertebral osteomyelitis	-	-	Oral amoxicillin for an 8-week course

Notably, our patient did not have signs of endocarditis on transthoracic echocardiogram (TTE), despite most cases of *S. cristatus* infections being reported in the setting of endocarditis. In the absence of suggestive clinical signs or bacteremia in his case, a transesophageal echocardiogram was not performed.

In most of the cases described, the diagnosis was made by bacterial culture (on blood, synovial fluid, vitreous fluid, and resected cardiac valve). The use of universal PCR targeting 16S rRNA on a resected pulmonary valve allowed for the diagnosis of endocarditis in one case (all cultures negative) [5]. Similarly, this technique allowed for the identification of this microorganism in a case of vertebral osteomyelitis for which blood cultures and biopsy cultures remained negative, in a patient sharing the same age as ours but with additional comorbidities [10].

Microbiological cultures are considered the gold standard for identifying pathogenic bacteria. However, the sensitivity of these cultures is highly variable and depends on the context in which samples are collected and on the pathogen involved. While the use of automatic blood culture bottle systems to incubate osteoarticular tissue samples has shown an improved and quicker detection of pathogens, false-negative cultures can still result from the presence of slow- or challenging-to-culture bacteria [22]. However, antibiotics administered before the sampling process remain the main cause of inconclusive cultures. Other limitations include the potentially limited quantity of material collected, and therefore the low quantity of microorganisms, and conditions during the pre-analytical phase (including duration before culture initiation). Therefore, culture fails to identify the etiological agent in one-third of pyogenic spondylodiscitis cases [23]. 

Molecular assays for infectious diseases have emerged as a new tool in clinical decision-making by identifying pathogens missed by conventional modalities. Numerous authors have previously demonstrated enhanced diagnostic accuracy through a universal PCR approach, targeting bacterial 16S rRNA [24-26]. Detection rates vary according to the type of infection, ranging from 50% to 70% for spondylodiscitis [23]. However, some authors have emphasized its lack of specificity (contamination causing false-positive results since even the smallest amount of DNA can be amplified), suggesting that its use be restricted mainly to culture-negative cases when infection is suspected [26,27].

Over the past few decades, several French authors investigated the benefits of using 16S PCR in the diagnosis of bone and joint infections [28-30]. The sensitivity of PCR combined with culture, ranging from 81.6% to 88.2%, was shown to be higher than culture alone, with a reservation regarding *Staphylococcal* infections in some studies showing no significant gain with 16S PCR, presumably due to poor lysis of *Staphylococci* cell wall at the DNA extraction step [28,29]. On the other hand, the specificity of 16S PCR combined with culture is expected to be lower than culture alone, mainly due to the amplification and detection of bacterial contaminants on the samples, even though precautions can be taken to effectively prevent the risk of contamination during the sampling procedure [28,29]. Among those studies, molecular methods not only allowed a quicker diagnosis than culture alone for fastidious bacteria but also produced bacteriological diagnoses when the conventional culture had failed [29]. In a larger French series of 3840 bone and joint culture-negative samples collected from 2308 patients suspected to have an osteoarticular infection, 16S PCR was positive in 9% of culture-negative specimens, and prior antibiotic therapy was identified as a potential explanation for the discrepancy between a positive PCR result and a negative culture in 31.4% of cases involving *S. aureus *[30].

Our case report takes part in the growing body of literature emerging from these advances. Between 2001 and 2007, the application of 16S rRNA sequencing led to the discovery of 215 newly identified bacterial species from human specimens, including 29 associated with novel genera [31]. We can, therefore, expect a growing number of diagnoses that were previously missed by conventional techniques.

The duration of antibiotics for spondylodiscitis has been a subject of debate but a French RCT showed that six weeks of antibiotic treatment was not inferior to 12 weeks [32].

The choice of empirical antibiotic therapy should take into account the most likely pathogens, the local epidemiology of resistance, the patient history, clinical presentation, and the presumed portal of entry. The lack of an identified pathogen poses several problems. First, there is the risk of treatment failure if an ineffective antibiotic is chosen. Second, the use of unnecessarily broad-spectrum antibiotics for an extended duration (six weeks) also exposes the patient to increased toxicity and contributes to the development of resistance. Molecular analysis can, therefore, be of significant help, but its inability to ascertain the susceptibility of the identified strains poses a challenge.

The inclination toward intravenous antibiotics is indicative of a widely accepted belief that parenteral therapy possesses an intrinsic superiority over oral therapy. However, this has recently been challenged by the OVIVA trial, which demonstrated that appropriately selected oral antibiotic therapy was noninferior to intravenous therapy when used during the first six weeks in the management of bone and joint infection [33]. Classical indications for surgery include failure of conservative management, failure of CT-guided sampling, mechanical instability, and compression of neurological structures. In our case, surgery was justified by a symptomatic neurological compression by the epidural abscess, thereby allowing early collection of abundant tissue samples from the infection site without the need for initial iterative percutaneous sampling, which is an unusual diagnostic approach.

## Conclusions

We report here the second case of spondylodiscitis due to *S. cristatus*, a microorganism that has been previously described in a few cases of bacteremia and endocarditis, although its virulence is still to be clarified. In both cases described to date, the identification of this pathogen required the use of 16S rRNA PCR sequencing, emphasizing its usefulness in the diagnosis of osteoarticular infections, especially when conventional cultures are inconclusive. In bone and joint infections such as spondylodiscitis, the identification of the causative microorganism remains crucial to confirming the diagnosis and selecting the best antibiotic strategy. However, the inability to ascertain the antibiotic susceptibility of the identified strains with molecular techniques poses a challenge.
